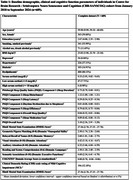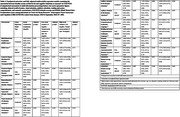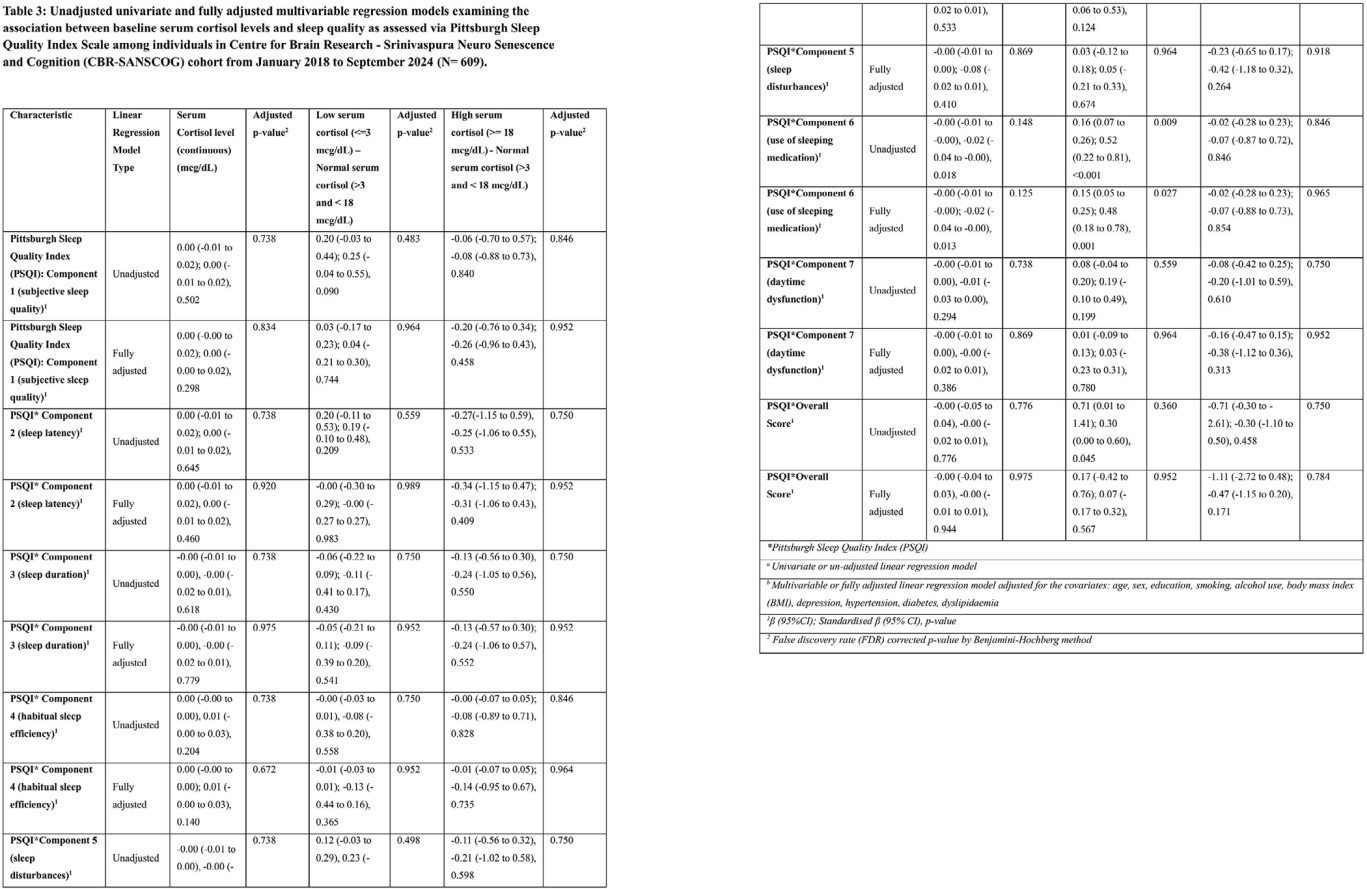# Cross‐Sectional Links between Baseline Serum Cortisol Levels and Cognitive Performance, Sleep, and Brain MRI Findings in a Rural Aging South‐Indian Cohort

**DOI:** 10.1002/alz70860_106975

**Published:** 2025-12-23

**Authors:** Sumedha Mitra, Jonas S Sundarakumar, CBR‐SANSCOG

**Affiliations:** ^1^ Centre for Brain Research, Indian Institute of Science, Bangalore, Karnataka, India

## Abstract

**Background:**

This study examined the relationship between baseline serum cortisol levels and cognitive performance in aging populations, an area with mixed findings in prior research. Some studies have linked higher cortisol levels with poorer cognition and lower brain volumes, while others found no association. However, this relationship remains unexplored in Indian populations.

**Method:**

Using cross‐sectional baseline data from 609 individuals (>=45 years age) in the CBR‐SANSCOG study, a large‐scale rural community‐based longitudinal aging study in South India, morning baseline serum cortisol levels were measured using electrochemiluminescence immunoassay. These levels were categorized into low (≤3 mcg/dL), normal (>3and<18 mcg/dL), and high (≥18 mcg/dL). Cognitive function was assessed globally and across various cognitive domains, using the Hindi‐Mental State Examination (HMSE) and COGNITO (Computerized assessment of adult information processing). Sleep quality was measured using the Pittsburgh Sleep Quality Index (PSQI). A subset of 123 participants underwent 3T MRI, and region‐specific white matter hyperintensity (WMH) volumes were extracted using FreeSurfer and LST‐AI toolbox. Linear regression models adjusted for age, sex, education, BMI, hypertension, diabetes, dyslipidemia, depression, smoking, alcohol use, and total intracranial volume (for MRI), were used to examine associations.

**Result:**

Findings revealed that individuals with low serum cortisol levels exhibited significantly poorer performance on the auditory attention task in COGNITO (β(95%CI)=‐1.22(‐2.19,‐0.26), *p* = 0.01, adj. *p* = 0.04) and reported increased use of sleep medication as reflected in higher scores in PSQI component 6 (β(95%CI)=0.15(0.05,0.25), *p* = 0.001, adj. *p* = 0.02). Cortisol levels were negatively associated with infratentorial WMH volume in males (β(95%CI)=‐0.00(‐0.01,‐0.00), *p* = 0.04, adj. *p* = 0.27) and positively associated with juxtacortical WMH volume in individuals below 60 years (β(95%CI)=0.03(0.00,0.06), *p* = 0.03, adj. *p* = 0.31), though these associations were not significant after false discovery rate correction.

**Conclusion:**

The findings suggest that lower serum cortisol levels, rather than higher levels as previously suggested, are associated with poorer cognitive performance in the attention domain and increased reliance on sleep medication. These results highlight the potential role of adrenal insufficiency, whether endogenous or exogenous, in cognitive function and sleep quality in aging populations. Further research is needed to explore this association by using diagnostic criteria to characterize adrenal insufficiency and investigating its impact on cognition and brain MRI volumes.